# Caregiving of Older Persons during the COVID-19 Pandemic in the Russian Arctic Province: Challenges and Practice

**DOI:** 10.3390/ijerph19052775

**Published:** 2022-02-27

**Authors:** Elena Golubeva, Anastasia Emelyanova, Olga Kharkova, Arja Rautio, Andrey Soloviev

**Affiliations:** 1 Department of Social Work and Social Security, Northern (Arctic) Federal University, Northern Dvina Embankment, 17, 163002 Arkhangelsk, Russia; e.golubeva@narfu.ru; 2 Thule Institute, University of Oulu & University of the Arctic, Paavo Havaksen Tie 3, P.O. Box 7300, FI-90014 Oulu, Finland; arja.rautio@oulu.fi; 3 Department of Psychiatry and Clinical Psychology, Northern State Medical University, 51, Troitsky Avenue, 163061 Arkhangelsk, Russia; harkovaolga@yandex.ru (O.K.); asoloviev1@yandex.ru (A.S.); 4 Arctic Health, Faculty of Medicine, University of Oulu, P.O. Box 5000, FI-90014 Oulu, Finland

**Keywords:** COVID-19, older person, caregiver, caregiver’s support, family-focused care, health, self-isolation, Russia

## Abstract

Older people and their families were particularly affected during the COVID-19 pandemic in 2020, but not much is known about the context of the Arctic regions of Russia. In this study, we identified the changes in family care before and during the pandemic using a questionnaire for the informal caregivers of older people. We investigated how and to what extent the pandemic has affected the relationships between caregiver and older person, and how the mental and physical health of older people and caregivers were affected by self-isolation in the Arkhangelsk region of Russia. The pandemic has changed the contribution of care from various actors: the share of care by charities, churches, and other aid agencies increased, while that of municipal services decreased. Sixteen percent of female and forty percent of male caregivers informed the study that COVID-19-related restrictions led to deterioration in the health of older people cared for at home. Family caregivers’ own health worsened, especially mental health: 28% of caregivers reported aggravated stress during the COVID-19 pandemic and expressed various fears. Our data show that the main resources in overcoming the period of self-isolation have been telephone communication, personal contact, reading/music, friends, as well as the help of social services and maintaining a positive attitude.

## 1. Introduction

The rapid spread of the COVID-19 pandemic around the world made it difficult to quickly assess its impact and predict its consequences. The fight against the emerging virus revealed multiple national problems in the fields of health care and social protection of people, as well as difficulties related to policy and economic mechanisms for regulating the epidemic situation. To curb the spread of the disease, countries adopted restrictive measures primarily aimed at reducing social contact. The restrictive measures included the temporary closure of public places, remote working (wherever possible), introduction of a pass system, and a mandatory mask regime. Russia introduced most of the restrictive measures in March 2020 on the basis of the “high alert regime” provided for by the Federal Law No. 68-FZ of 21 December 1994 “On the Protection of Population and Territories from Natural and Technogenic Emergencies” [1]. Restrictions were introduced in many regions of the country; however, they were partly different from each other. Those restrictions imposed by the federal government were gradually supplemented and expanded by regional governments. The strict self-isolation regime was imposed on older people aged 65 and over, prohibiting them from leaving their homes in the Arkhangelsk region.

According to the all-Russian survey of the National Agency for Financial Research, almost every third Russian family has members of old age who need constant care and attention (30%), and in most cases they are cared for by their relatives. This situation is most acute for residents of small cities, towns, and villages (35–40%). The overwhelming majority of Russian citizens take care of their older relatives on their own (94%) [2]. Nowadays, the topics of coronavirus, its consequences, and the effects of restrictive measures have been growing in the academic literature. Specific recommendations and reports for one of the most vulnerable population groups, “older people”, have been released at the international, national, and local levels with regard to the diagnosis, treatment, and management of COVID-19 in older people [3]. Already a substantial body of research has studied, for example, the efficacy and safety of COVID-19 vaccines in older people [4,5], and social isolation in households and long-term care facilities [6,7,8,9].

In Russia, there is as yet scattered and limited research about the impact of the pandemic and governmental restrictive measures on social institutions, society, and various population groups. There are published studies, for example, on health care clients [10,11,12] and hired personnel [13,14].

In Russia, with the spread of the COVID-19 virus, the regime of long-term self-isolation for older people, who were most susceptible to psycho-social problems triggered by the spread of the pandemic, was accompanied by the rather negative dynamics of their somatic status and mental health. In addition, active media campaigns about the threats of a pandemic and the regime of self-isolation have changed people’s usual way of life, exacerbating the negative experiences associated with the fear of contracting COVID-19. Kelasev and Pervova noted that the attitude of older people to the media is very different: from complete faith to the harshest criticism [15]. Parfenova also highlighted intimidation from the television and a lack of verified and reliable information, which undoubtedly contributed to the stress of the older population during the coronavirus pandemic [16].

The aim of our research was to identify the impact of the COVID-19 pandemic on the caregivers and care needs of older people in the Arctic province of Russia, with the Arkhangelsk region as an example. There has been little research conducted in the Arctic territories of Russia, even though COVID-19 underscores existing vulnerabilities of Arctic communities and may produce new challenges, especially for older people as a risk group [17]. Arctic communities have unique health and social needs and face distinctive circumstances, e.g., due to complex high-level mortality owed to chronic underlying health conditions and infectious diseases, such as tuberculosis. They also have longstanding severe physical and social infrastructure deficits, inadequate access to health care, and fragile and/or resource-based economies [18].

Our specific research questions included: Has the pandemic affected the relationship between at-home caregivers and older people during the period of self-isolation? Have there been any changes in the needs of services, time spent in care, and personal relationships before and during the pandemic? What third-party or external resources have helped the caregivers during the self-isolation period? Has the pandemic led to a deterioration in the mental and physical health of older people and their caregivers during the period of self-isolation?

## 2. Materials and Methods

To achieve the aims and answer the research questions stated, we used a questionnaire for informal caregivers of older people. After the onset of retirement age at 55 years for women and 60 years for men in the Arctic territories of the Russian Federation, people obtain the right to access social services. Due to the difficulty of reaching the target group independently, as well as restrictions related to the pandemic, the questionnaire was spread face-to-face with the help of local social services (social workers), who help families caring for older people. Social workers belonged to six social service centers located in the cities of Arkhangelsk, Kotlas, and other community centers in the Arkhangelsk region. It was not possible to use social media and other online channels because the majority of study participants do not use them. The caregivers of older people already living in nursing homes, as well as caregivers who were not family members, were excluded from the sample. It was not possible to compensate for the participation in the study as this research was not funded at the moment of data collection.

The participants signed a written consent form. The sample size consisted of 90 caregivers, who cared for an older relative entitled to receive social services at home and voluntarily agreed to participate in the research study: 15 men and 75 women aged 22 to 74 years old, average age 46.4 ± 11.6 years old.

The data collection was organized and conducted in spring 2020, during the first wave of the coronavirus pandemic. At that time, there were restrictive federal and regional governmental actions and measures in the Arkhangelsk region of Russia. A self-isolation regime was imposed on people over 65 and people suffering from chronic diseases, in addition to the requirements to wear a mask in all public places and keep a distance from other people [19]. The Governor of the Arkhangelsk region signed a decree on the introduction of a preparedness regime as well as on measures to counter the spread of coronavirus infection in the area [20]. Later, that decree was updated several times to regulate procedures for extending the effect of restrictive measures.

To determine the challenges in the care of older people, we used the published questionnaire developed by Prof. Zs. Szeman from Semmelweis University, Institute of Mental Health (Hungary) [21]. It focuses on aspects of the caregiver–care recipient relationship (and pandemic-related changes in this relationship); caregiver’s responsibilities; caregiver’s self-reported physical, general, and mental health status, including the level of stress experienced due to the pandemic; the type and source of help the caregiver received with care-related activities, both before and after the declared state of emergency; the types of physical and mental resources that caregivers were able to utilize in order to cope with the difficulties posed by the pandemic; and problems in carrying out care tasks and the factors that helped. The questionnaire was presented in its original form (in Russian) to Russian respondents [21]. We used Pearson’s Chi-squared tests with standardized adjusted residuals and Mann–Whitney U tests were used. The latter were carried out using SPSS Statistics (version 24.0.0.0, IBM, Armonk, NY, USA). Differences were considered statistically significant when the level of statistical significance (*p*) was less than 0.05.

## 3. Results

### 3.1. The Pool of Studied Caregivers

From the 90 respondents, 75.5% were relatives of the care clients (child 43.3%, another relative 23.3%, grandchild 8.9%) and 24.5% of the informal caregivers were acquaintances (neighbors, friends).

Most of the interviewed caregivers (93.3%) lived in the city or district center where the social service center is located, and 6.7% of caregivers were from remote villages without any social service center in the proximity. There were 62 people (68.9%) who resided separately from an older person in care, and 28 people (31.1%) who lived together with them. Around 27% of respondents have provided care for a period of 1 to 6 months, and 73% were involved for more than a year.

The average social status of the sampled caregiving relative is characterized by the highest attained educational level and their occupation. The sampled caregivers had secondary vocational education (50%), higher university education (43.4%), and secondary education (6.6%). The occupational levels were public sector workers, medical workers, and civil servants (48.9%), trade/commerce workers and entrepreneurs (20%), manual workers (12.2%), not working or retired (12.2%), and in a leadership/managerial position (6.7%).

According to the regional governmental guidelines on the provision of social services in the Arkhangelsk region, social workers provide the following types of social services in cooperation with relatives or informal caregivers: household; medical; psychological; pedagogical; communication (informing on different topics of interest, encouraging e.g., physical activity); employment-related; and legal services for the recipients of social services [22]. It is known that older people may live separately from their families or share a common household, but the desire to live independently from families is closely associated with the ability of the state care system to meet their needs, as well as with the cost of such services for older people or their families [23]. Before the COVID-19 pandemic, 68.9% of respondents lived separately from their relatives in need of care, while 31.1% lived together.

### 3.2. The Analysis of the Input from Various Care Actors

**Figure 1:**
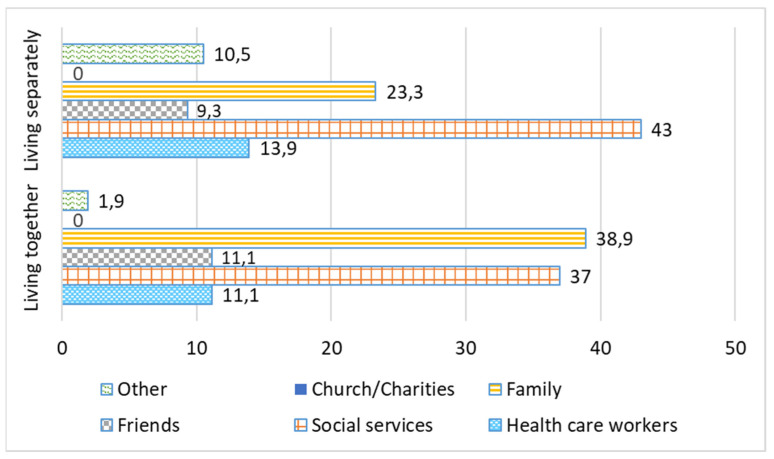
Before the introduced restrictions of the COVID-19 pandemic: the input of various care actors. Each type of residence adds up to 100% within its own group: “Living separately” and “Living together”, %.

**Figure 2:**
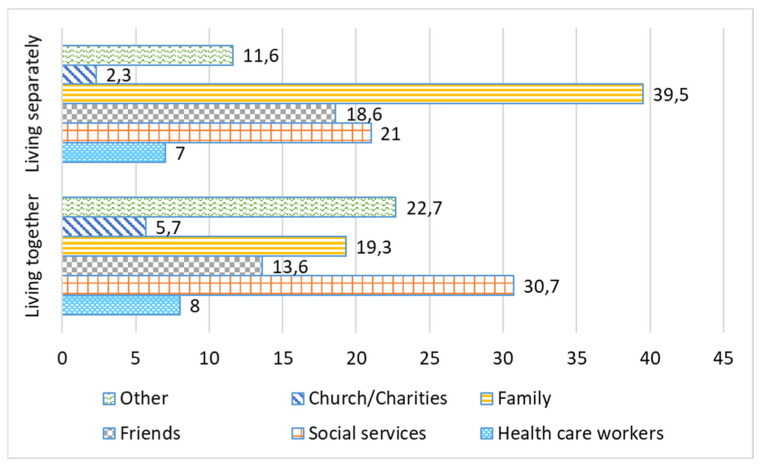
During the restrictions of the COVID-19 pandemic: the input of various care actors. Each type of residence adds up to 100% within its own group: “Living separately” and “Living together”, %.

According to our research, older people had various relationships with family, friends, and neighbors. The ideal situation for older people is having close social ties, but a sufficiently high level of independence. There is a need for a sensible combination of family care and personal autonomy [24]. Figure 1 and Figure 2 show the need for care services from various actors before and during the COVID-19 pandemic. According to Figure 1, before the pandemic restrictions when the caregiver and an older care recipient lived separately, a greater amount of care was received from social services (43%). In the case of living together with a care recipient, more responsibilities of care naturally fell on the family’s shoulders and, respectively, the share of received social services from other actors was lower.

During the imposed pandemic restrictions, the role of other aid agencies noticeably increased, replacing the part of public services. The latter has decreased for both types of residence—when a caregiver resides together with and separately from the care receiving older person—by 13% and 16%, respectively, during the pandemic (Figure 2). Volunteer associations, charitable organizations, and other non-governmental organizations (NGOs) showed successful examples of assistance practices during the COVID-19 pandemic, helping the self-isolated older people.

Comparative analysis in care provision before and during the pandemic showed a decrease in assistance of 5.2% in formal health care and 13.2% in social services during the period of the imposed restrictions. There was also a 3.3% decrease in family care during the COVID-19 pandemic. Presumably this was due to social distancing, self-isolation of older people, and a lack of personnel at social and health care institutions. At the same time, we noticed an increase in care during the pandemic provided by friends, neighbors, acquaintances (5.3%), and charitable organizations (16.4%).

### 3.3. Gender Aspects and Duration of Care Responsibilities before and after the Pandemic Restrictions

There were practically no differences in the range of services provided to older people when living separately and together with a caregiver. However, there were some gender differences when older care-recipients communicated with a caregiver (*p* = 0.032) (Table 1).

The average duration of care before the introduction of COVID-19 pandemic-driven restrictions in the entire sample of respondents was 4.3 h in one day. In total 30% of the respondents believed that the length of care had increased since the onset of the pandemic. A total of 10% of the respondents said they have less time to provide care. The remaining 60% believed that there were no significant changes in the time spent for caregiving.

Table 2 specifies the time spent on caregiving by the sex of a caregiver. Based on the obtained data, most female caregivers informed us that the time spent on care during the period of restrictions had not changed (66.7%). On the contrary, 25.3% of females informed us that the time spent providing care during the COVID-19 pandemic had increased, and 8% answered that they did not have as much time as before and the time for care had decreased. In the subgroup of male care providers, the majority (53.3%) increased the time spent caring for their older relative and 20% estimated that the time decreased. The remaining number of respondents (26.7%) in the subgroup of men informed us that the time spent on caregiving had remained unchanged (Table 2).

**Figure 3:**
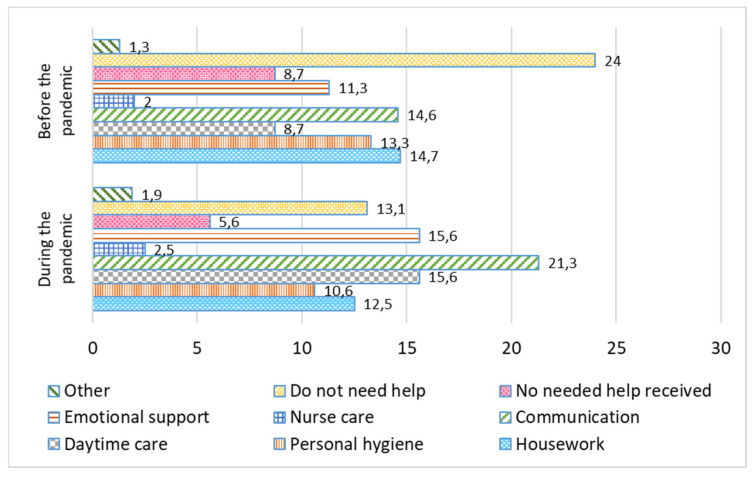
Needs for different types of assistance before and after the introduction of COVID-19 restrictions, %.

Social restrictions during the pandemic have led to changes in the range of services available in the care sector. Before the pandemic, 24% of families did not need any help, 14.6% of families needed help with additional communication and information services, and 14.7% needed help with household work. After the introduction of restrictions, the need for communication services increased from the initial 14.6% to 21.3%. A smaller percent of families informed us that they did not need any help after the pandemic started: only 13.1%, compared to 24% of families before the pandemic (Figure 3).

### 3.4. Caregiver’s Health Status

The results showed that in families where women provided care, the health condition of 16% of the care-receiving older people worsened, while the rest of the respondents did not observe any significant changes in the health of care recipients (Table 3). In the subgroup of male caregivers, 40% of the older people experienced a worsening of health conditions during the pandemic in terms of chronic diseases, an increase in body temperature, a decline in mental health presenting as a refusal to communicate, and feelings of fear and anxiety. The explanation for this may be that caregiving men offered less emotional support and communication with an older person.

### 3.5. Pandemic-Related Restrictions and Relationships between Older Persons and Caregivers

**Figure 4:**
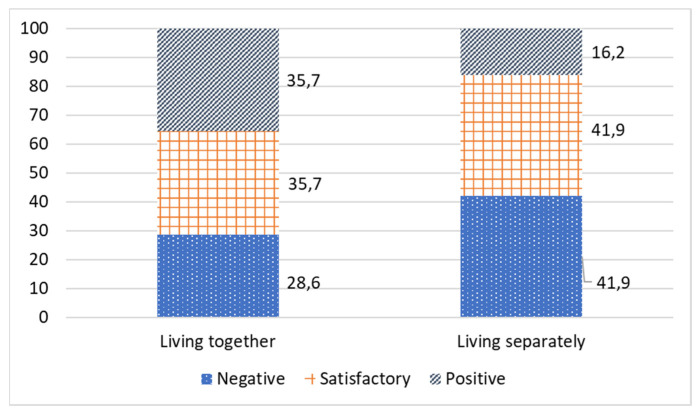
Comparative analysis of the relationship of a caregiver with an older relative during self-isolation, depending on residence type, %.

It has been rather difficult to combine family care and personal autonomy during the pandemic after imposing the self-isolation regime. Around 9% of respondents moved to live with their relative in need of care (Figure 4), and this has affected their relationship.

According to Figure 4, the majority of respondents in the studied sample assessed their relationship with an older relative as satisfactory. When the caregiver and older person lived separately, 41.9% of caregivers had a satisfactory relationship. This dropped to 35.7% when the caregivers lived together with an older care recipient.

During the self-isolation period, positive relationships between a caregiver and older person have been much stronger if they live together in one household (35.7% vs. 16.2% when living separately). There was a positive dynamic in the relationships during the period of self-isolation for those living together, and a negative impact of the pandemic restrictions for those who lived separately (Table 4).

**Figure 5:**
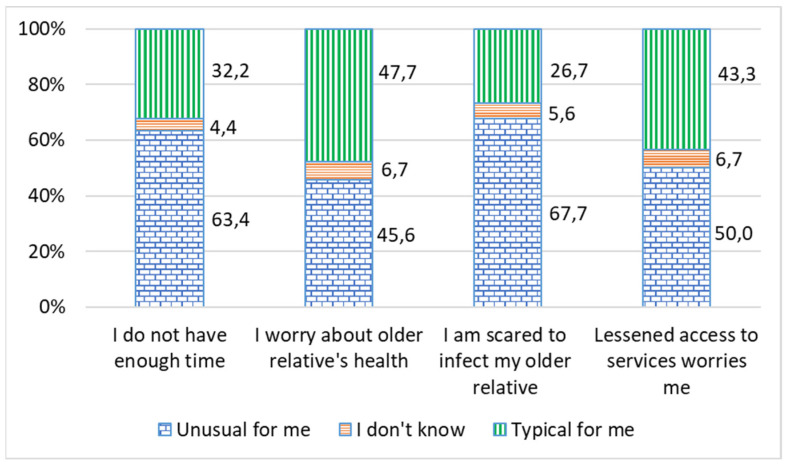
Fears of the caregivers during self-isolation during the COVID-19 pandemic, %.

As many as 28% of caregivers reported aggravated stress during the COVID-19 pandemic. The biggest concerns were the health of their older relative (47.7%), reduced access to care services (43.3%), and a fear of infecting their relative (26.7%) (Figure 5). The family caregiver living together with an older care recipient showed higher values on the anxiety scale (U = −2647, *p* ≤ 0.002) [25].

### 3.6. External Resources Helping Caregivers to Cope during the Self-Isolation Period

Self-isolation and limited contact were reported as the biggest problems for people caring for their older relative during the COVID-19 pandemic (35.7%). There were also problems in the acquisition of personal protective equipment (15.3%), e.g., the shortage and excessively expensive cost of masks, gloves, and antiseptics. In addition, the respondents reported negative reactions to those who did not follow recommendations (mask/glove use, distancing, a large amount of people gathering in one place (11.2% respondents)). The emergence of such problems was associated with an increase in the time spent caring for a sick relative (8.2%), problems with medical supervision (8.2%), and the emergence of feelings of fear and anxiety (7.1%).

Among 90 respondents, 81% reported that telephone communication helped to survive the period of restrictive measures during the pandemic, as well as personal contact (71.2%), reading/listening to music (65.6%), and friends (62.2%) (Table 4).

According to Table 5, for caregivers, the main resource in managing the difficult situation caused by the restrictions was the help of a social worker and social service organizations (28.2%). Emotionally, a positive attitude that the pandemic would soon end and life would return to its usual course helped caregivers remain resilient (15.4%). The next resources were also helpful during the self-isolation period: volunteer assistance to deliver groceries, online shopping, communication, and entertainment (each 10.3%).

The assistance of specialists from social and health care institutions remains in demand under any circumstances: when an older person in need of care and a family caregiver live together, or separately, and both before and during the period of the spread of coronavirus infection. A relative or another caregiver undoubtedly needs additional help from the outside, especially those who continue to combine work and care. Because of restrictive measures, many older people were unable to satisfy even their basic needs, such as adequate nutrition and maintaining health, due to limited access to public places. Caring for the older person in the pandemic-imposed conditions proved more difficult.

## 4. Discussion

Our findings are in agreement with the significant pandemic-related stress factors for older adults noted by Bubeyev et al.: (1) a sharp change in professional status (for the older generation, the most significant is the loss of professional employment and income); (2) changes in the structure and content of significant social roles of the individual in the field of professional employment and family relations (lack of social and material benefits, as well as situations, where constant care and assistance of a relative is needed). As a result, diseases and episodes of personal crisis develop; (3) deformation of significant social ties that provoke strong emotional experiences and are perceived by the individual as major life failures; (4) the experience of a “hostile environment” and the manifestation of aggression towards people around them (the perception of others as potential carriers of the virus and a probable source of illness and death) [26].

Evidence shows that care by family members remains a cultural norm in Russia, and that is why the burden on caregivers has been increasing. In our research study 75% of all caregivers were women. The fact that caring for older relatives is more often the responsibility of women is also found consistently in the reports from other countries [27]. Researchers also highlight the risks in family care practices and policy: the obligation to provide care for older family members can often push women out of the labor market [28]. Inadequate levels of care allowances can also increase their risk of poverty. If family care becomes more and more widespread in society, it will reduce the responsibility of the state and can create difficult situations for the family [29]. At the same time, care work does not receive sufficient institutional support [30]. Several authors note that family care is perceived as the fulfillment of a moral duty. On the other hand, there may be negative aspects of practicing family care: a decrease in the quality of life, an increase in risks to the physical and psychological health of the caregiver, and the possible lack of help from other actors [23,30,31,32]. Mental health deterioration has been widely noted in the research on the health status of caregiving people. Increased anxiety occurs e.g., due to inadequate understanding or ignorance of how to provide care correctly, and what state support is available. Irritability, depression, and turning to psychotropic drugs are the main mental health problems in caregivers [27]. Due to the lack of awareness, family members lose their resilience, the level of their financial and legal vulnerability increases, and social isolation occurs not only to sick older people but also to family members and loved ones who take part in their care [33]. The results of our study add that self-isolation restrictions during the pandemic exacerbated the above stated risks, and they worsened, especially the health of both parties in the family care and also the relationships when trapped in the place of residence.

Increased fear and anxiety about one’s future was a response to the introduction of a long-term self-isolation regime for the older age population, accompanied by the negative dynamics of somatic diseases and mental health problems in these people [26]. The most negative expectations of the COVID-19 pandemic consequences were found, first of all, in people aged 65+ from the risk group with comorbid somatic diseases: arterial hypertension, heart disease, diabetes, and obesity [26].

Family care has its own potential risks, costs, and consequences, but the combined efforts of the state and the family respond more quickly to the changing needs of an older person in various services. There is the predominant responsibility of the family in care and the well-being provision for older relatives, a gender-skewed profile of care (it is mainly the responsibility of women), the lack of institutional recognition [30], as well as lack of social and health care assistance to older people in need. These risks manifested themselves most vividly in the situation of pandemic restrictions and self-isolation. At the same time, one of the priorities of the Russian Strategy of Action for the Benefit of Older People [34] is to build an effective system for the provision of social services, based on the individual needs of citizens, contribution and support from families, and the possibility of receiving at-home social services for older people with dementia without placing them into social service institutions.

We found that before the COVID-19 restrictions, Russian churches and charitable organizations were typically invisible in the system of social care for older people (0% before the pandemic), but their role increased to 2–6% during the pandemic. Non-profit organizations have united at the national and regional levels, offering various assistance services in more than 60 regions of the Russian Federation, for example the delivery of food and drugs, online courses for computer literacy, and others. Dobrokhleb found that older people preserved their relations with family, friends, and neighbors during the pandemic, and people began to support each other even more actively than before [35].

The obtained results may be of interest to regional and municipal social services responsible for supporting the older population, especially in the framework of preventive measures for the next epidemic wave. We conclude that the key factors to overcome the COVID-19 pandemic-related negative consequences for older people are the solidarity of generations, manifested in family cohesion, development of the volunteer movement, proper care, access to information, as well as state support for ensuring the rights of older people.

Our survey of 90 caregivers showed that the self-isolation regime during the COVID-19 pandemic noticeably affected the relationship between at-home caregivers and older people. For instance, 54% of male caregivers and 25% of females increased the time spent caring for their older relative. The pandemic has also changed the input from various actors, e.g., the share of care by charities, churches, and other aid agencies increased, and public (municipal) services decreased. At the same time, the need for communication and information services increased by 6.7% during the self-isolation period compared to pre-pandemic. This implies a need for more available information concerning which NGOs are available to help during this specific pandemic time, and where and how to contact them.

The COVID-19-related restrictions have deteriorated the health condition of older people cared for at home (reported by 16% of female and 40% of male respondents of our survey). The respondents’ own health worsened as well, especially mental health: 28% of caregivers reported aggravated stress during the COVID-19 pandemic and expressed various fears. These are important findings with practical implications, such as additional support to tackle the symptoms of anxiety or depression for caregivers as well as older care recipients.

## 5. Conclusions

We noted that there was a tendency for positive relationships during the period of self-isolation for people living together and a negative impact of the pandemic restrictions for those who lived separately. This may call for additional state measures to encourage a co-habiting way of care during a period of self-isolation whenever possible in each individual pair of caregiver–care recipient. The main resources in overcoming the period of restrictions during the pandemic were telephone communication, personal contact, reading/listening to music, friends, as well as the help of a social worker, social service organizations, and maintaining a positive attitude. Possible governmental support could be, for example, provision or advice on unlimited and flexible monthly cell and landline phone plans; educational assistance in learning new ways to spend time at home; access to the Internet, and more entertainment and communicational channels.

Our findings are in line with earlier studies, which found that the need for care increases with age due to deteriorating health. The main burden of care falls on women (75% caregivers in our study were women). Women are especially engaged in care if a significant amount of time is required (more than 20 h a week). Such intensive care reduces employment opportunities for caregivers, worsens their health, and it can cause a lack of time to take care of their own health. In addition, significant physical stress arises from caring for people with limited mobility in old age or with disabilities. Families that live with an older relative, who needs care, should have had help from social and medical services during the COVID-19 pandemic. However, there was a significant reduction in the assistance provided by social and medical institutions. This imbalance must be addressed and corrected by the government at different levels.

More research is needed to determine the interplay between pandemic-related self-isolation and the relationship and health of at-home caregivers and older people, as well as the resources needed for providing such care efficiently. In addition, future research should explore the interaction of these results with the severity of the pandemic, which differs between various regions of the Arctic, especially in the vast Arctic of Russia, and hence is expected to have different consequences. Lastly, we should note that our findings refer to spring 2020, the first COVID-19 phase after the outbreak. It would be beneficial to learn of further impacts to the older population, which may be aggravated by longer periods of self-isolation in the following waves of the pandemic.

## Figures and Tables

**Table 1:** Differences by sex in the caregiving responsibilities *.

Responsibilities	Sex, N, Percentage		*p*-Value
	Females, N = 75	Males, N = 15	
Feeding	34, 45%	7, 47%	0.925
Bathing	25, 33%	6, 40%	0.620
Dressing	17, 23%	3, 20%	0.821
Diaper changing	17, 23%	5, 33%	0.380
Transferring	16, 21%	6, 40%	0.125
Medication administration	48, 64%	11, 73%	0.487
Cooking	46, 61%	10, 67%	0.697
Shopping	55, 73%	11, 73%	1.000
Housework/laundry	39, 52%	7, 47%	0.706
Handling administrative, official affairs	39, 52%	10, 67%	0.298
Housework	29, 39%	6, 40%	0.923
Social interactions	43, 57%	13, 87%	0.032
Other	3, 4%	1, 7%	0.647

* Note: N—number of respondents that marked the responsibility as relevant to them (e.g., feeding: 34 females feed their older person under care and the remaining 41 do not). The N is followed by % (the percentages of each responsibility and sex add up to 100%); *p* was calculated with the Pearson χ2 test.

**Table 2:** Change in time providing care based on sex (%) *.

Time Spent on Caregiving	Sex, N, Percentage		*p*-Value
	Females, N = 75	Males, N = 15	
Increased	19, 25%	8, 53%	0.015
Did not change	50, 67%	4, 27%	
Decreased	6, 8%	3, 20%	

* Note: N—number of respondents by sex followed by %; *p* was calculated using the Chi-square test.

**Table 3:** Changes in the health status of a care-receiving older relative during the pandemic, depending on who provided care (by sex) *.

Was There a Deterioration in the Health of an Older Relative during the Pandemic	Sex, N, Percentage		*p*-Value
	Females, N = 75	Males, N = 15	
Yes	12, 16%	6, 40%	0.004
No	60, 80%	6, 40%	
I don’t know	3, 4%	3, 20%	

* Note: N—number of respondents by sex followed by %; *p* was calculated with the Pearson χ2 test.

**Table 4:** Types of resources helping the caregiver to cope with COVID-19 pandemic-related problems.

Resource Types	My Resource *	Not My Resource
Online liturgy/church services	100	0
Phone connection with family	81.1	18.9
Personal contact with family	71.2	28.8
Phone connection with friends	65.6	34.4
Personal contact with friends	62.2	37.8
Internet connection with family	42.2	57.8
Internet connection with friends	36.7	63.3
Reading/listening to music	33.3	66.7
Internet, cinema, theater	27.8	72.2
Cooking	24.4	75.6
Gardening	23.3	76.7
Tourism/sport	15.6	84.4
Praying	12.2	87.8
Online studying	11.1	88.9
Online chatting	8.9	91.1

* Each resource adds up to 100% between the two groups: “My resource” and “Not my resource”.

**Table 5:** Types of resources used by a caregiver during the pandemic.

Resource Types	% in Descending Order *
Help from a social worker and social services	28.2
Positive attitude	15.4
I have the capacity to provide care and do not need help	10.3
Online shopping, communication, entertainment	10.3
Help from volunteers, e.g., grocery delivery	10.3
Help from other family/friends	8.9
Personal hygiene assistance	5.1
Availability of free time	5.1
Availability of needed information	3.8
Limitation of contacts	2.6
Others	0.9

* All resources add up to 100%.

## Data Availability

Not applicable.
